# Enhancement of the bioavailability of a novel anticancer compound (acetyltanshinone IIA) by encapsulation within mPEG-PLGA nanoparticles: a study of formulation optimization, toxicity, and pharmacokinetics

**DOI:** 10.18632/oncotarget.14481

**Published:** 2017-01-04

**Authors:** Qi Wang, Na Wei, Xiaofeng Liu, Alex Chang, Kathy Qian Luo

**Affiliations:** ^1^ School of Chemical and Biomedical Engineering, Nanyang Technological University, Singapore; ^2^ Department of Oncology, Johns Hopkins Singapore, Singapore; ^3^ Faculty of Health Sciences, University of Macau, Taipa, Macau, China

**Keywords:** anticancer drugs, acetyltanshinone IIA, bioavailability, mPEG-PLGA, toxicity, pharmacokinetics

## Abstract

The Poly (ethylene glycol) methyl ether-block-poly (lactide-co-glycolide) (mPEG-PLGA) nanoparticles carrying acetyltanshinone IIA (ATA), a novel anti-breast cancer agent, were prepared by ultrasonic emulsion method to enhance the bioavailability and reduce the toxicity. Systematic optimization of encapsulation process was achieved using an orthogonal design. Drug efficacy analysis showed that ATA nanoparticles were as effective as free ATA against estrogen receptor positive breast cancer cells, but much less toxic towards human endothelial cells. Furthermore, in zebrafish, ATA nanoparticles displayed much lower toxicity than free ATA. More importantly, the blood concentration of ATA nanoparticles indicated by 24 hour-area under the curve (AUC_0-24h_) was 10 times higher than free ATA. These results indicated the potential of ATA-loaded mPEG-PLGA nanoparticles for the delivery of ATA in a clinical formulation, and their potential for use in tumor therapy in the future.

## INTRODUCTION

Worldwide, breast cancer is the predominant cause of female mortality. Among all types of breast cancers, approximately 65% express high levels of the estrogen receptor (ER) [1–3]. ER is a transcription activator that usually localized in the nucleus in an inactive state [4–12], and is activated mainly by estrogen. Currently, the most effective drug to treat ER+ breast cancer are tamoxifen and fulvestrant. However, the inhibitory effects of the commercialized drugs on ER+ cancers are still far away from satisfying because of the drug resistance and partial therapeutic responses [13–17].

Tanshinone IIA (TIIA), a lipophilic pharmacologically active compound extracted from the medicinal herbal plant Radix *Salviae miltiorrhizae* (Danshen), has been widely used to treat cardiovascular diseases [18–20]. In addition, recent pharmacological studies have revealed the anti-cancer effect of TIIA on various types of cancer cells [21–22]. Recently, we found that a chemically modified compound of TIIA, acetyltanshinone IIA (ATA), had the potential to be a more effective anti-ER+ breast cancer agent than the current therapeutics [23].

First, ATA exhibited stronger growth inhibition of ER+ breast cancer cells than tamoxifen [23]. Second, although both ATA and fulvestrant could bind to ER and cause it to degrade, ATA completely abolished the presence of ER while fulvestrant only reduced the protein level [23]. Third, ATA reduced the expression of ER at the mRNA level, while fulvestrant did not [23]. Finally, ATA reduced the transcription of a major ER-responsive gene, GREB1, indicating an ability to repress the transcription activity of ER [23]. These merits suggest that ATA is a promising anti-ER+ breast cancer candidate for pharmaceutical development. However, our previous study in rats indicated low bioavailability for ATA. To solve this problem and prepare for future clinical trials of ATA, a therapeutically applicable formula of ATA that can improve *in vitro* aqueous solubility and *in vivo* bioavailability was developed.

Conventional preparation methods, such as solution, suspension, and emulsion, fail to provide sustained therapeutic effects owing to limitations such as low availability, intolerance, and instability. Compared to these conventional methods, nanoparticles offer higher stability, larger capacity, and a controlled release profile. After considering various encapsulation strategies, poly(ethylene glycol) methyl ether-block-poly (lactide-co-glycolide) (mPEG-PLGA) was selected for the encapsulation of ATA because it exhibits higher bioavailability and a longer circulation period [24–26]. More importantly, both PEG and PLGA have been approved by the United States Food and Drug Administration for medical applications. Herein, we report the generation, characterization, *in vitro*/*in vivo* validation, and pharmacokinetic study of ATA-loaded mPEG-PLGA nanoparticles (ATA NPs). The improved solubility and bioavailability of ATA NPs demonstrated that mPEG-PLGA is an ideal material to encapsulate ATA. Furthermore, this formulation can potentially be used in future clinical studies of the anticancer efficacy of ATA.

## RESULTS

### Chemical synthesis of ATA

**Figure 1:**
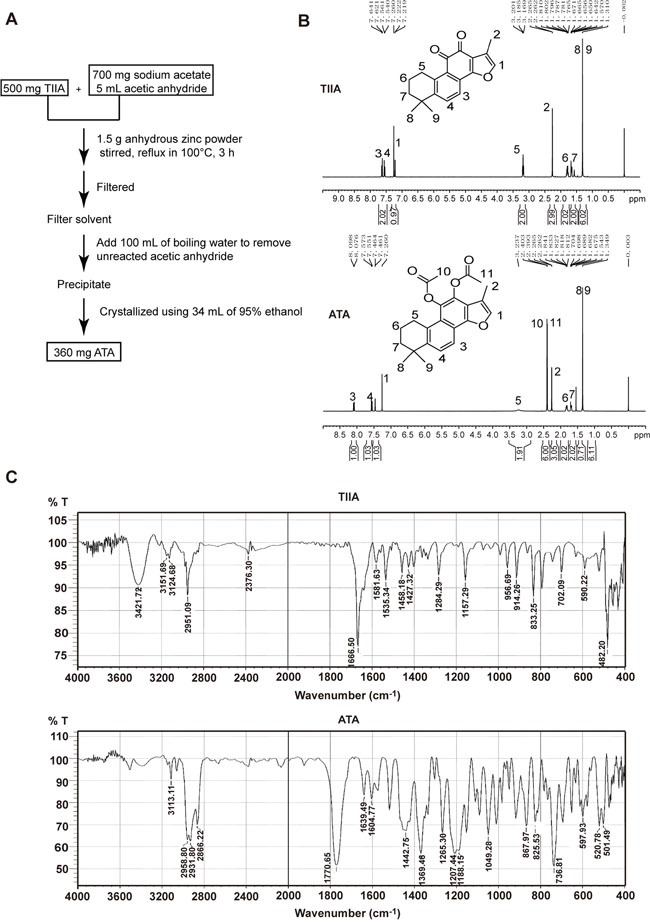
Synthesis of ATA **A**. Processes of ATA synthesis. **B**. ^1^H NMR spectra of TIIA and ATA. **C**. FITR spectra of TIIA and ATA.

ATA was synthesized by the reduction and modification of the two carbonyl bonds of TIIA into two ethyl ester bonds using sodium acetate, acetic anhydride, and zinc. Boiling water was utilized to remove unreacted acetic anhydride through a hydrolysis reaction, and the final product was obtained by purification through recrystallization in 95% ethanol (Figure 1A) [22]. The recovery rate of ATA was 72%.

^1^H NMR analysis was used to determine the structure of ATA. Figure 1B shows the ^1^H NMR spectra of TIIA and ATA. The following proton signals of TIIA (1: –CH at δ 7.22; 2: –CH_3_– at δ 2.27; 3,4: –CH– at δ 7.64 and 7.56; 5: –CH_2_ at δ 3.19; 6,7: –CH_2_– at δ 1.79 and 1.64; 8,9: –CH_3_ at δ 1.31) were observed in the ATA molecule. The characteristic signals of 10 and 11 at δ 2.39 attributed to –CH_3_– were from ATA. These new signals indicated the appearance of two ethyl ester bonds formed by the attachment of acetic anhydride to the carbonyl group. This NMR analysis indicated the successful synthesis of ATA.

Fourier transform infrared (FT-IR) spectra further confirmed the successful synthesis of the compound ATA. Figure 1C displays the FT-IR spectra of TIIA and ATA. A characteristic band of TIIA was detected at 2951.09 cm^−1^, which was assigned to the C-H vibration. A large peak was also observed at 1666.50 cm^−1^, which was assigned to the C=O group. The characteristic bands of ATA, which were assigned to the C-H group, occurred at 2958.80 cm^−1^, 2931.80 cm^−1^, and 2866.22 cm^−1^. A large band was also observed at 1770.65 cm^−1^, which was assigned to the C=O group of ATA. In a comparison between TIIA and ATA, the C-H signal of ATA was stronger and contained more divided peaks than TIIA, because of the increased number of C-H bonds and more complicated environment in ATA. However, the C=O signal in TIIA was affected by the aromatic ring, so the peak position (1666.50 cm^−1^) was smaller than the C=O signal in ATA (1770.65 cm^−1^). Finally, the conjugated system of TIIA is weaker than ATA, resulting in fewer, and weaker, C=C stretching signal peaks around 1600 cm^−1^.

### Optimization of critical factors for formulating ATA NPs

Before designing the orthogonal array, several preliminary experiments were carried out to determine the important factors and their approximate effective ranges. Then, an orthogonal experimental design was conducted to design nine experiments [L9(3^4^)] to test three factors (A: percentage of F68, B: ratio of mPEG-PLGA:ATA, and C: ultrasonic time) at three levels. The experimental design is shown in Table 1 and the results are shown in Table 2. Based on the results, a suitable formulation using the best normalized percentage of encapsulation efficiency (EE%) and drug loading (DL%) was selected.

**Table 1:** Orthogonal array test design with three variable factors and levels

Parameter designation	Variables	Variable levels		
		Level 1	Level 2	Level 3
A	Percentage of F68 (%)	0.2	0.5	1.0
B	Ratio of mPEG-PLGA:ATA (m:m)	15	20	25
C	Ultrasonic time (min)	1.5	2.0	2.5

**Table 2:** Results of the orthogonal array tests

Test number	Factors			Encapsulation efficiency (%)	Drug loading (%)
	A (F68%)	B (Ratio of mPEG-PLGA:ATA)	C {Ultrasonic time (min)}		
1	0.2	15	1.5	83.91	5.30
2	0.2	20	2.0	90.95	4.35
3	0.2	25	2.5	84.10	3.25
4	0.5	15	2.0	89.34	5.62
5	0.5	20	2.5	93.42	4.46
6	0.5	25	1.5	100.29	3.86
7	1.0	15	2.5	89.81	5.65
8	1.0	20	1.5	86.47	4.14
9	1.0	25	2.0	100.10	3.85

The EE% and DL% obtained from different experimental design runs are shown in Table 2; all experimental runs were performed three times. The optimized formulations in Table 2 are Test 6 and Test 9, which have an EE% of 100.29% and 100.10%, and DL% of 3.86% and 3.85%, respectively.

Based on statistical analysis of the data, each factor was ranked according to its influence on the EE% and DL% of the ATA NPs (Table 3). The influence on EE% of ATA decreased in the order A>B>C, as indicated by the R values of the orthogonal L9(3^4^) test. The maximum EE% of ATA was obtained when the F68 percentage was 0.5%, the mPEG-PLGA: ATA ratio was 25:1, and the ultrasonic time was 2.5 min (i.e., A2, B3, C3 in Table 1). The influence on DL% of ATA decreased in the order B>A>C, as indicated by the R values of orthogonal L9(3^4^) test. The maximum DL% of ATA was obtained when the F68 percentage was 0.5%, the mPEG-PLGA: ATA ratio was 15:1, and the ultrasonic time was 2.5 min, respectively (i.e., A2, B1, C3 in Table 1).

**Table 3:** Analysis of orthogonal array tests

		Factors		
		A (F68%)	B (Ratio of mPEG-PLGA:ATA)	C {Ultrasonic time (min)}
Encapsulation efficiency (%)	k1	86.32	87.69	90.22
	k2	94.35	90.28	89.11
	k3	92.13	94.83	93.46
	R	8.04	7.14	4.36
	Sequence of significant effect of factors	A>B>C		
	Optimal condition	0.5% of F68	mPEG-PLGA:ATA (m:m) = 25:1	Ultrasound for 2.5 min
Drug loading (%)	k1	4.30	5.52	4.43
	k2	4.64	4.31	4.45
	k3	4.54	3.65	4.60
	R	0.34	1.86	0.17
	Sequence of significant effect of factors	B>A>C		
	Optimal condition	0.5% of F68	mPEG-PLGA:ATA (m:m) = 15:1	Ultrasound for 2.5 min

*k1, k2, k3: Average at variable levels

* R: Representative range

With respect to the influence of the three tested factors on EE% and DL%, the B factor showed more influence on the DL% than the EE% of ATA NPs. Considering the experimental results, the B1 level (mPEG-PLGA:ATA=15:1) was favored to prepare the ATA NPs, and thus the final composition of A2B1C3 was adopted. Although this final composition was chosen in favor of the DL% of ATA NPs, nonetheless, a very good EE% of 87.69% was achieved.

### Characterization of ATA NPs

**Figure 2:**
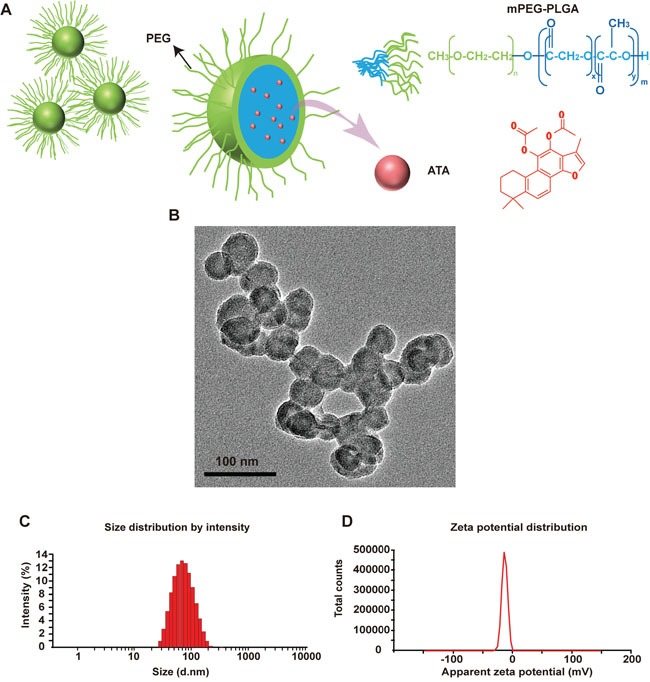
Characteristics of ATA NPs **A**. Scheme of the structure of ATA NPs. **B**. TEM of ATA NPs. **C**. Size of ATA NPs. **D**. Zeta potential of the ATA NPs.

The low water solubility of ATA had previously restricted usage *in vivo*. To improve solubility, reduce toxicity, prolong circulation time, and enhance bioavailability, ATA NPs were formulated as shown in Figure 2A. The ATA molecules were encapsulated in the hydrophobic core of NPs, with the hydrophilic chains of PEG facing outside to avoid phagocytosis by microphages. The ATA NPs were prepared according to the optimized preparation method.

We then determined essential physicochemical properties of the ATA NPs including morphology, size, charge, and physical state. Transmission electron microscopy (TEM) analysis confirmed that ATA NPs were spherical (Figure 2B) and measurements from the Malvern Nano-ZS Particle Sizer indicated an average size of 69.93 nm (Figure 2C). The zeta potential of ATA NPs was -14.7 mV (Figure 2D), which indicated a satisfactory surface charge distribution that is important for the stability of NPs. The optimized ATA NPs exhibited a drug EE% of 88.10% and DL% of 5.54%.

**Figure 3:**
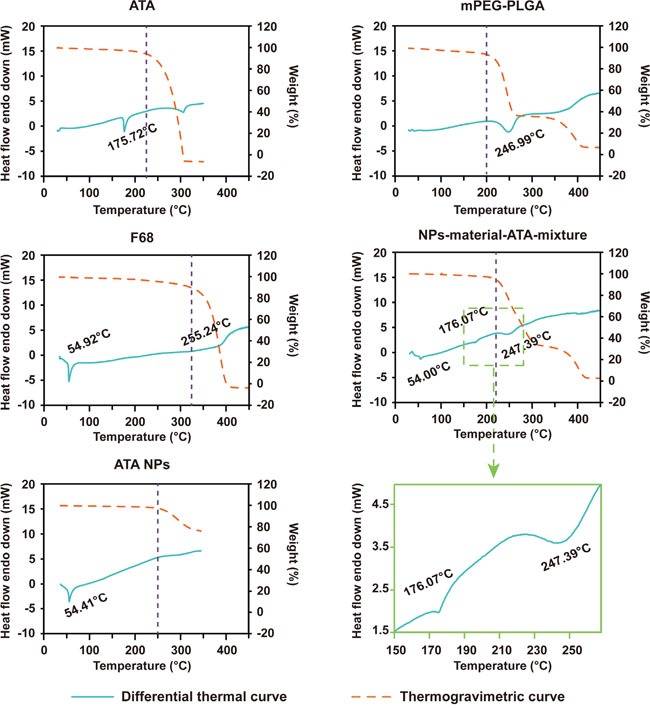
TG/DTA profiles of ATA, mPEG-PLGA, F68, the mixture of ATA, mPEG-PLGA and F68, and ATA NPs

The thermal analysis of ATA NPs was conducted using thermogravimetry (TG) and differential thermal analysis (DTA). The results of pure/unformulated and formulated ATA are presented in Figure 3. The TG curve of pure ATA showed that the mass loss of ATA occurred in just one step within the temperature range of 220-310°C. This step consisted of accelerated mass loss reaching ~100%, which was associated to a small DTA endothermic peak. The DTA curve showed the appearance of an endothermic peak at 175.72°C which was caused by the phase transition of ATA. The TG profile of mPEG-PLGA is different from that of ATA, as the mass loss occurred in three steps: step 1 in the temperature range of 200-260°C, step 2 in 260-350°C and step 3 in 350-400°C. It was apparent that mPEG-PLGA did not have a phase transition peak in the DTA curve before mass loss. Similar to pure ATA, the TG curve of F68 displayed one step of mass loss, but in the higher temperature range of 320-400°C with a small DTA endothermic peak. The phase transition temperature of F68 was at 54.92°C, which is much lower than that of free ATA (Figure 3).

In the analysis of the physical mixture of the three components of ATA NPs: ATA, mPEG-PLGA, and F68, we found that the mass loss occurred after the temperature crossed 220°C. Three DTA peaks were found at 54.00°C, 176.07°C and 247.39°C, which corresponded to the phase transition temperature of F68 (54.92°C in the F68 curve), ATA (175.72°C in the ATA curve), and mPEG-PLGA (246.99°C in the mPEG-PLGA curve), respectively. In contrast to the physical mixture, the curve for ATA NPs showed only one phase transition temperature (54.41°C), which suggested that ATA was molecularly dispersed within the matrix of NPs, and demonstrated the amorphous nature that further confirmed the encapsulation of ATA by the matrix (Figure 3).

### Evaluation of the stability and drug release profiles of ATA NPs

**Figure 4:**
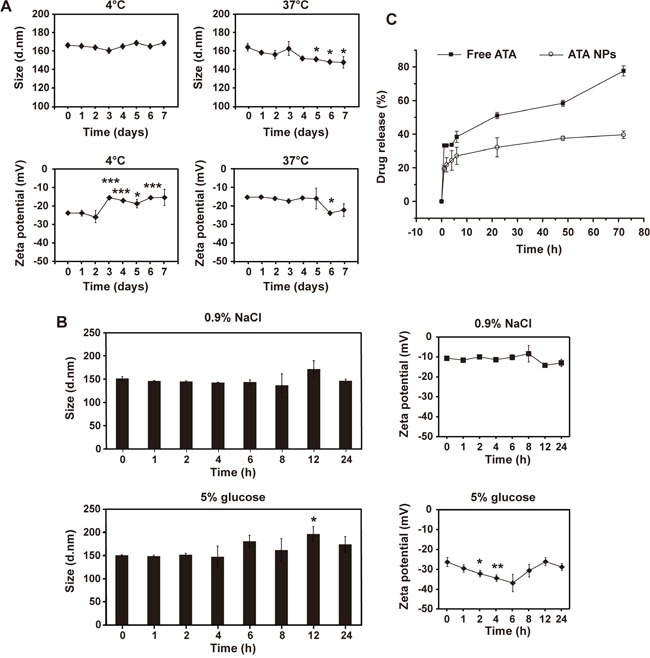
Stability of ATA NPs **A**. The size and zeta potential of ATA NPs in PBS at 4°C or 37°C after storage for 1-7 days.*p < 0.05, **p < 0.01, ***p < 0.001 compared with day 0. **B**. The size and zeta potential of ATA NPs in 0.9% NaCl or 5% glucose at 25°C for 24 h. *p < 0.05, **p < 0.01, ***p < 0.001 compared with 0 h. **C**. Drug release profile.f2 < 50, comparison between free ATA and ATA NPs group.

The storage stability of ATA NPs under various conditions was compared by monitoring changes in size distribution and surface charge. At 4°C, although the zeta potential of ATA NPs showed some variations after 3 days of incubation, the size of ATA NPs in PBS did not change significantly over a period of seven days (Figure 4A). In contrast, both the particle size and zeta potential were significantly decreased after storage in PBS at 37°C for 5-7 days. These results suggested that ATA NPs were stable in PBS at 4°C for seven days and at 37°C for four days.

Solutions of 0.9% NaCl and 5% glucose were compared for the compatibility-stability study of ATA NPs at room temperature for 24 h. The results presented in Figure 4B show that no significant changes of size and zeta potential were detected in 0.9% NaCl. In contrast, when ATA NPs were incubated with 5% glucose at room temperature for 24 h, their size varied at 12 h and their zeta potentials were significantly reduced at multiple time points between 2 h and 6 h (Figure 4B). These results indicate that ATA NPs are more stable in 0.9% NaCl than in 5% glucose at room temperature, and should therefore be prepared in 0.9% NaCl for use in future clinical applications.

Next, we compared drug release profiles of ATA NPs with those of free ATA. Owing to the hydrophobic nature of ATA, the resulting poor solubility in aqueous media led to a low concentration gradient across the dialysis membrane, which slowed down the passive diffusion process (Figure 4C). However, ATA NPs displayed even slower drug release profiles than free ATA within the 72 h-tested periods. For example, after 22 h of drug release, 50% of ATA was released from free ATA solution, while only 30% was released from ATA NPs. A similar trend of slower release was also observed from ATA NPs at the later time points of 48 and 72 h (Figure 4C). In addition, unlike free ATA, the release profile of ATA NPs shows no burst release of ATA at earlier time points (Figure 4C), indicating that ATA was almost completely entrapped in the NPs with few free ATA molecules in solution or absorbed on the surface of NPs. In summary: encapsulation of ATA with mPEG-PLGA significantly prolonged its release from the NPs in this study.

### *In vitro* determination of the anticancer efficacy and general toxicity of ATA NPs

As ATA was previously shown to prominently inhibit growth of ER+ breast cancer cells [23], MCF-7 breast cancer cells that express a high level of ER were used to evaluate the anticancer efficacy of ATA NPs. Meanwhile, human umbilical vein endothelial cells (HUVEC) were used to determine the general toxicity of ATA NPs on non-cancerous cells. Cells were treated with free ATA or ATA NPs at six concentrations: 0.625, 1.25, 2.5, 5, 10, and 20 μM for 24, 48, and 72 h. Cell viability was determined by the MTT assay and the experiment was conducted in triplicate.

**Figure 5:**
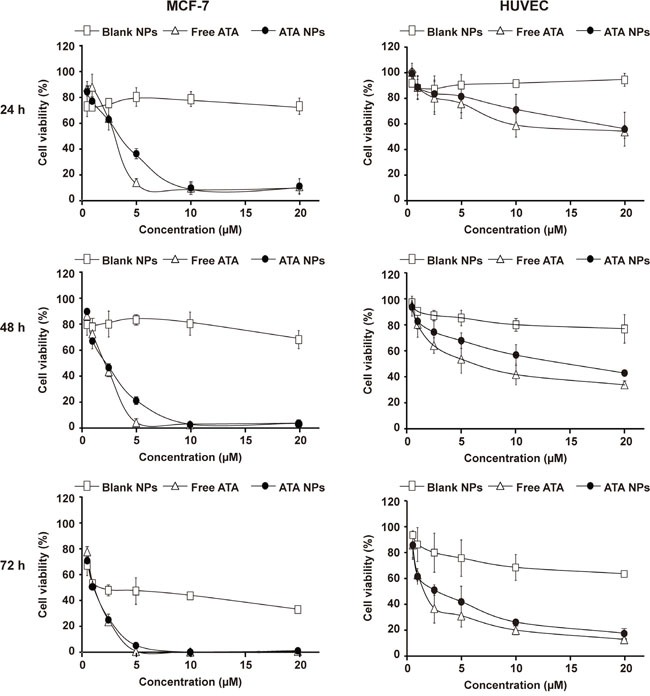
Cytotoxicity of free ATA in solution and ATA NPs against MCF-7 breast cancer cells and HUVEC The cytotoxicity was determined by the MTT assay for 24, 48, and 72 h.

The dose-response curves in Figure 5 show that at high concentrations of 10 μM and 20 μM, both free ATA and ATA NPs effectively reduced the viability of MCF-7 cells by approximately 90% at 24 h, and by almost 100% at 48 and 72 h. Although the efficacy of 5 μM ATA NPs is lower than that of free ATA at 24 h, this difference was reduced at 48 h and was further diminished at 72 h. More importantly, the half-maximal inhibitory concentration (IC_50_) values of free ATA and ATA NPs in MCF-7 cells were very similar at all three time points (Table 4) and ATA-NPs even achieved a slightly lower IC_50_ value at 72 h of 0.99 μM compared with free ATA at 1.06 μM. Overall, these results demonstrated that encapsulation of ATA in the NPs did not decrease its anticancer efficacy compared with free ATA on ER+ breast cancer MCF-7 cells; thus, the ATA NPs formulation successfully retained the original anticancer efficacy of ATA.

**Table 4:** IC_50_ values of ATA NPs *vs* free ATA

		IC50 (μM)		IC50 ratio
		Free ATA	ATA NPs	
MCF-7	24 h	2.62	2.72	1.04
	48 h	1.72	1.96	1.14
	72 h	1.06	0.99	0.93
HUVEC	24 h	19.32	31.45	1.63
	48 h	6.67	13.53	2.03
	72 h	2.05	2.76	1.35

*IC_50_ ratio: IC_50_ of ATA NPs/IC_50_ of free ATA.

Compared with the significant cytotoxicity of ATA on breast cancer MCF-7 cells, both forms of ATA were much less toxic to normal HUVEC cells (Figure 5). In particular, the 24 h-IC_50_ value of free ATA in HUVEC cells (19.32 μM) was 7.4-fold higher than the value in MCF-7 cells (2.62 μM) (Table 4). More interestingly, the 24 h-IC_50_ values of ATA NPs in HUVEC cells (31.45 μM) was even higher (11.56-fold) than the value in MCF-7 cells (2.72 μM). These results clearly demonstrate that the cytotoxic effects of ATA NPs are more specific in cancer cells than normal cells.

### Evaluation of the impact of ATA NPs on the development and heart rate of zebrafish

**Figure 6:**
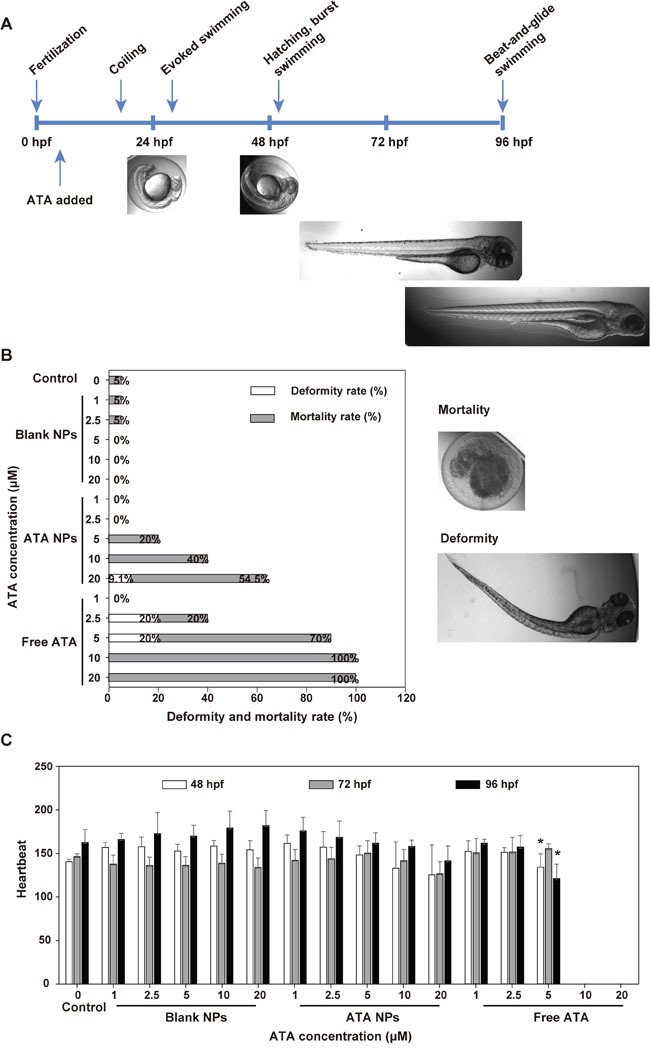
Toxicity of ATA NPs in zebrafish **A**. Zebrafish morphology in the first 96 hpf. **B**. Deformity and mortality rate of zebrafish (n=80-120 fish). **C**. Heartbeat of zebrafish. *p < 0.05, **p < 0.01, ***p < 0.001, comparison between free ATA and the control group.

Zebrafish have been proven to be a rapid, cost-effective model for toxicity studies. Here, they were used to evaluate the *in vivo* toxicity of ATA NPs as zebrafish embryos finish their development in four days (Figure 6A). Various concentrations of the free and nanoparticle forms of ATA were added to zebrafish embryos at four hours post fertilization (hpf). The subsequent morphological changes were observed at 96 hpf and heartbeats were measured between 48-96 hpf. Images of the normal morphology of zebrafish larvae from 0 to 96 hpf are shown in Figure 6A, while the examples of dead and deformed larvae are shown in Figure 6B (right side).

Between 20-30 fish larvae were used for each condition and each experiment was performed for four times. Figure 6B shows the overall percentages of deformed and dead zebrafish in each group. The data from Figure 6B demonstrate that free ATA in the concentration range of 5-20 μM caused high toxicity in zebrafish development. In particular, 100% mortality was recorded in fish treated with 10 μM and 20 μM free ATA for 96 hpf, and 70% mortality plus 20% deformity was observed at 5 μM. Even at 2.5 μM, free ATA damaged zebrafish by causing a total of 40% death and abnormal development. These results suggested that dosages of free ATA higher than 1 μM caused deformity or mortality in zebrafish larvae.

In contrast, ATA NPs demonstrated significantly lower toxicity in zebrafish larvae than free ATA. For example, much lower mortality rates were recorded when fish larvae were treated with ATA NPs at 5, 10, and 20 μM for 96 hpf compared to free ATA. Additionally, no zebrafish mortality was observed from ATA NPs at the concentrations of 1 μM and 2.5 μM, which is 2.5-fold higher than the 72 h-IC_50_ value in breast cancer MCF-7 cells. This result indicates that ATA NPs have lower *in vivo* toxicity than free ATA.

In addition to that evaluation of larval mortality and abnormality, we also monitored the zebrafish heart function. As concentrations of 10 μM and 20 μM free ATA killed all the fish larvae within 96 hpf, no heartbeats were recorded with these two drug concentrations (Figure 6C). Treatment with 5 μM free ATA significantly reduced the heartbeats recorded at 96 hpf from 160 beats per minute (bpm) in normal fish larvae to 120 bpm in ATA NPs-treated fish larvae (Figure 6C). No signs of cardiotoxicity were observed from fish larvae treated with ATA NPs at 1-10 μM for 72 hpf (Figure 6C). These results further confirmed our finding that the nanoparticle formulation of ATA is safer than free ATA.

### Determination of the pharmacokinetic profiles of ATA NPs in rats

#### Validation of the analysis methods for determination of ATA concentration in rat plasma and blood

**Figure 7:**
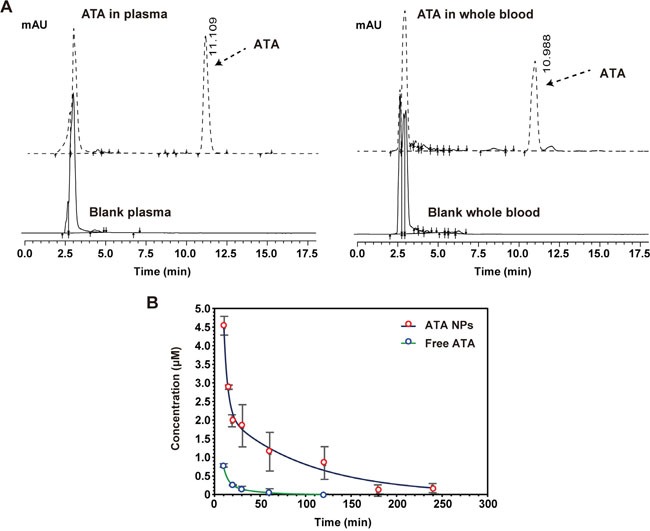
PK profile of the ATA NPs **A**. Endogenous interferences on rat plasma and blood. **B**. PK profile of free ATA and ATA NPs in rat blood.

Under the optimized chromatographic conditions, ATA was well separated in rat plasma and whole blood samples with retention times of 11.109 and 10.988 min, respectively. Additionally, there were no obvious endogenous interferences under the described chromatographic condition within the retention times of 4.0-17.5 min in both rat plasma and blood (Figure 7A).

The calibration curve was prepared by linear fitting of the peak area of ATA versus the standard solutions. Table 5 lists the regression equation, coefficients of determination (R^2^), the detection limit and quantitation limit. The high R^2^ values indicated that the regression equations for analyzing ATA were statistically acceptable.

**Table 5:** Regression equation of ATA in rat plasma and whole blood (n=5)

	Concentration range (μM)	Regression equation	R2	Detection limit	Quantitation limit
Plasma	0.37−7.37	Y=43572X+3986.1	R^2^=0.9959	0.018 μM	0.37 μM
	7.37−27.63	Y=51717X-15943	R^2^=0.9925		
	27.63− 644.74	Y=54955X+4.5*10^5^	R^2^=0.9937		
Whole blood	0.37−7.37	Y=52900X-2183.1	R^2^=0.9990	0.37 μM	0.37 μM
	7.37−27.63	Y=59871X-16042	R^2^=0.9970		
	27.63−644.74	Y=76891X-4.9*10^5^	R^2^=0.9930		

*Y: area of the peak

* X: concentration (μM)

The extraction recoveries of ATA from spiked rat plasma and blood are listed in Table 6. The lowest extraction recovery of ATA for all tested concentrations was 80.42% in the plasma and 81.77% in the blood, respectively. No matrix effect on the analysis of ATA was obvious after these treatments.

**Table 6:** Extraction recoveries of ATA from rat plasma and whole blood (n=5)

	Added (μM)	Determined (μM)	Extraction recovery (%)	RSD (%)
Plasma	0.37	0.29±0.03	80.42±8.76	10.90
	27.63	28.32±4.29	102.46±15.52	15.15
	644.74	642.71±0.89	99.68±0.14	0.14
Whole blood	0.37	0.29±0.03	81.77±5.32	6.51
	27.63	26.05±3.24	94.34±11.70	12.40
	644.74	648.50±21.68	100.58±3.37	3.35

The results of the precision and accuracy are shown in Table 7. The relative standard deviations (RSDs) of intra-day variation for ATA from spiked rat plasma and blood were less than 15.15% and 12.40%, respectively. On the other hand, the RSDs of inter-day variation for ATA from spiked rat plasma and blood were less than 7.03% and 5.11%, respectively. These results indicate that the current method had a satisfactory precision for the pharmacokinetic study of ATA NPs.

**Table 7:** Intra-day and inter-day precision of ATA in rat plasma and whole blood by HPLC (n=5)

	Theoretical concentration (μM)	Intra-day precision			Inter-day precision		
		Detected (μM)	Relative recovery (%)	RSD (%)	Detected (μM)	Relative recovery (%)	RSD (%)
Plasma	0.37	0.29±0.03	80.42±8.76	10.90	0.24±0.03	67.44±4.29	6.35
	27.63	28.32±4.29	102.46±15.52	15.15	25.82±1.82	93.38±6.56	7.03
	644.74	642.71±0.89	99.68±0.14	0.14	573.79±10.03	88.99±1.56	1.75
Whole blood	0.37	0.29±0.03	81.77±5.32	6.51	0.29±0.00	78.36±3.25	3.79
	27.63	26.08±3.24	94.34±11.70	12.40	23.87±1.21	86.40±4.41	5.11
	644.74	648.50±21.68	100.58±3.37	3.35	613.39±12.92	95.14±2.00	2.10

### Determination of the pharmacokinetic profile of ATA NPs in rats

The validated RP-HPLC method was used to determine the concentration of ATA in rat plasma and whole blood following a single intravenous administration of free ATA and or ATA NPs at 8 mg/kg body weight, for both forms. As the ATA concentration in plasma was too low to be detected after 15 min (possibly due to absorption by red blood cells), we instead measured ATA concentration in whole blood. The average whole blood concentration of ATA was plotted against the time of sample collection and displayed in Figure 7B. Compared with free ATA, ATA NPs displayed prolonged circulation time during the 240-min test (*p* < 0.05). The blood ATA concentration in the free ATA-treated group declined rapidly, and reached a very low level after 30 min of ATA administration. In contrast, the blood ATA concentration in the ATA NPs-treated group declined at a much slower rate, and was still detectable 240 min after sample injection. In particular, the concentration of ATA was reduced to 0.5 μM in 12 min in the free ATA-treated group, while reduction to 0.5 μM did not occur until 150 min in the ATA NPs-treated group.

The pharmacokinetic parameters of ATA were determined using two-chamber model and calculated using Excel PKsolver. According to the requirements for the selection of pharmacokinetic calculation model, the values of Akaike's information criterion and residual sum of squares (Re) were minimized, while the value of the determinate coefficient (r) was maximized. The mean retention time was calculated by statistical moment analysis. The main pharmacokinetic parameters of free ATA and ATA NPs in rat whole blood are listed in Table 8.

**Table 8:** Pharmacokinetic parameters of free ATA and ATA-NPs in rat whole blood

Parameter	Unit	Free ATA	ATA NPs	Ratio of NPs/free ATA
k10	1/min	0.13	0.08	0.62
k12	1/min	0.05	0.13	2.60
k21	1/min	0.05	0.03	0.60
t1/2 alpha	min	3.55	3.03	0.85
t1/2 beta	min	22.84	63.78	2.79
C0	μM	4.06	26.20	6.45
V	(mg/kg)/μM	1.97	0.31	0.16
CL	(mg/kg)/μM/min	0.26	0.02	0.08
V2	(mg/kg)/μM	2.15	1.27	0.59
CL2	(mg/kg)/μM/min	0.10	0.04	0.40
AUC_0-24h_	μM·min	30.56	308.41	10.09
AUC_0-inf_	μM·min	30.87	324.67	10.52
AUMC	μM·min^2	490.97	20,766.87	42.30
MRT	min	15.90	63.96	4.02
Vss	mg/kg/μM	4.12	1.58	0.38

k10: elimination rate; k12: inter-compartment rate; k21: intra-compartment rate; t1/2 alpha: distribution half-life; t1/2 beta: elimination half-life; C0: initial concentration; V: apparent volume of distribution; V2: compartment volume of distribution; CL: clearance; CL2: clearance between central and peripheral compartment; AUC(0–24h): area under the plasma concentration-time curve from time zero to the last measurable concentration (24 h); AUC(0–inf): area under the plasma concentration-time curve from time zero to infinity; AUMC: area under the first moment curve; MRT: mean retention time from time zero to the last measurable concentration; Vss: Steady state volume of distribution.

The information in Table 8 further confirmed that encapsulation of ATA in NPs improved the bioavailability in rats. For example, the 24 h-total area under the curve (AUC_0-24h_) was increased 10-fold from 30.56 μM·min in free ATA to 308.41 μM·min in ATA NPs. Additionally, the mean retention time (MRT) of ATA NPs was four times longer than free ATA (63.96 min *vs* 15.90 min). These results showed that ATA NPs significantly increased the bioavailability of ATA in rats.

## DISCUSSION

In the previous study, our group demonstrated that acetyltanshinone IIA (ATA), a novel anti-cancer agent, can effectively inhibit the growth of oestrogen receptor positive (ER+) breast cancer cells. However, the low aqueous solubility limited the use of ATA because of the low bioavailability. Hence a method to improve its aqueous solubility and bioavailability is essential. In addition, chemotherapeutic drugs are known to have various side effects to normal organs and cells. This presents a need to find a way to reduce the toxic effects of ATA on healthy tissue.

In this study, ATA was synthesized based on our previously described method [22] and verified by NMR and FITR (Figure 1). mPEG-PLGA was selected as the encapsulation material for ATA into the NPs. mPEG-PLGA can protect the encapsulated content from being engulfed by phagocytic cells, and has become a biodegradable polymer that is widely used for drug delivery. To optimize the formulation of ATA-loaded mPEG-PLGA NPs, orthogonal design, a statistical method for analyzing several independent factors in a limited number of experiments, was utilized. The final ATA NPs were prepared using the optimized conditions of 0.5% F68, 2.5 min of ultrasonic time, and an mPEG-PLGA to ATA ratio of 15:1 (Table 3).

The resulting ATA NPs achieved a high encapsulation efficiency of 88.10% and a good drug-loading rate of 5.54%. The encapsulated ATA molecules were indicated by the TG/DTA analyses to be molecularly dispersed within the NP matrix which confirmed the entrapment of ATA (Figure 3). Moreover, the stability assays showed that ATA NPs remained stable after storage in a physiological saline solution at 4°C and 37°C for 5-7 days. In addition, the release of ATA was significantly prolonged by NP encapsulation.

One important advantage of the encapsulation of therapeutic agents into NPs is that it may reduce the potential toxicity of the active ingredients to normal cells. The *in vivo* toxicity evaluation of ATA NPs showed that the NP formulation produced much lower toxicity on the mortality, development, and heart function of zebrafish compared to the free ATA (Figure 6).

Finally, we developed a sensitive analytical method for measuring the plasma and blood concentration of ATA in rats. This method displayed a good linear relationship, improved extraction recoveries, and satisfied the required precision and accuracy for the pharmacokinetic study of ATA (Tables 5-7). The results showed that encapsulation of ATA in NPs significantly extended the circulation time of ATA during the test period (Figure 7B). The total area under the curve (AUC_0-24h_) and mean retention time (MRT) of ATA NPs in blood were notably increased compared to free ATA (Figure 7B and Table 8).

In summary, our study resulted in the successful preparation of ATA-loaded mPEG-PLGA NPs with satisfactory properties including valuable EE%, favorable size, and high stability. The drug efficacy of ATA NPs was equal to free ATA *in vitro*, and the ATA NPs conferred lower toxicity to normal cells and zebrafish and also prolonged the circulation time of ATA in rat blood. To our knowledge, this is the first study to use nanomization for the improvement of the bioavailability of the novel anticancer compound ATA. This study suggests that the nanoparticle encapsulation of ATA could offer a potential strategy to deliver ATA in clinical cancer therapy.

## MATERIALS AND METHODS

### Materials

mPEG-PLGA (mPEG, Mn 2,000; PLGA, Mn 4,000; LA:GA=1:1), Poloxamer 188, Sodium dodecyl sulfate (SDS), Tween 80, dialysis sacks (Mw cut-off: 12,000 Da) and 3-(4,5-dimethylthiazol-2-yl)-2,5-diphenyltetrazolium bromide (MTT) were purchased from Sigma-Aldrich (St. Louis, MO). Chloroform was obtained from Merck Pte. Ltd (Singapore).

Dulbecco's Minimum Essential Medium (DMEM), penicillin/streptomycin (PS), and trypsin were purchased from GIBCO. Fetal bovine serum (FBS) was purchased from Hyclone. HUVEC and MCF-7 breast cancer cells were obtained from the ATCC (Manassas, VA).

### Synthesis of ATA

TIIA (500 mg), sodium acetate (700 mg), and anhydrous zinc powder (1.5 g) were mixed with acetic anhydride (5 mL). The mixture was stirred and refluxed at 100°C for 3 h, and then filtered. The filtrate was diluted with water (100 mL) and boiled to remove excess acetic anhydride. The resulting solution was cooled and the precipitate was filtered again. This precipitate was recrystallized twice using 95% ethanol (34 mL) to produce the final product (approximately 360 mg)[22]. Proton nuclear magnetic resonance (^1^H NMR) spectra were recorded at room temperature with a Bruker spectrometer operating at 500 MHz and using DMSO-d6 as solvent. Fourier transform infrared (FT-IR) spectra were recorded with a Perkin-Elmer Spectrum 100 at a resolution of 2 cm^−1^.

### Preparation of ATA NPs

#### Systematic optimization using orthogonal design

The encapsulation efficiency of ATA and drug loading were selected as evaluation indices of the orthogonal design [29–31]. Three major effective factors {A: concentration of F68 (%) (B): ratio of mPEG-PLGA: ATA (m:m), and C: ultrasonic time (min)} associated with this process were optimized using an orthogonal L_9_(3^4^) test design as shown in Table 1. These three factors were tested at three different levels, and encapsulation efficiency and drug loading of ATA were chosen as the evaluation indices.

#### Encapsulation efficiency determination

ATA NPs (1 mL) were centrifuged at 500 rpm at 25°C for 5 min. A fixed amount of supernatant was diluted with methanol to the desired concentration and the concentration of ATA in the solution was referred to as the concentration of encapsulated ATA. The concentration of ATA was measured by RP-HPLC analysis (Shimadzu LC-20AT pump liquid chromatograph; chromatographic column: Kromasil 100-5C8, 250 mm × 4.6 mm, 5 μm) [32–36]. A methanol-H_2_O (80/20, v:v) mobile phase system was pumped at a flow rate of 1 mL/min with a column temperature of 30°C, and the column eluents were monitored at a wavelength of 254 nm. The percentage of drug loading (DL%) and encapsulation efficiency (EE%) was calculated as follows:

DL% = amount of drug in NPs / amount of feeding polymer

EE% = amount of drug in NPs / amount of feeding drug

#### Preparation of ATA NPs

Emulsion-solvent evaporation method was used to prepare the ATA NPs. First, ATA (2.5 mg) and mPEG-PLGA (37.5 mg) were dissolved in chloroform (500 μL). The resultant solution was then added into 0.5% F68 (10 mL) which was dissolved in PBS and treated with ultrasound (37% strength) for 2.5 min. Finally, chloroform was removed by rotary evaporation to obtain ATA NPs. Blank NPs were prepared by the same method, but without the addition of ATA into the formulation.

### Characteristics of ATA NPs

#### Size distribution and zeta potential

The size distribution and zeta potential of the nanoparticles were determined by laser electrophoretic light scattering analysis (Malvern Nano-ZS Particle Sizer).

#### Transmission electron microscopy (TEM) to determine the morphology of NPs

TEM analysis was used to determine the morphology of NPs. Briefly, an aqueous dispersion of NPs was placed on a formvar-coated copper TEM grid and allowed to dry. Excessive liquid was drained off using filter paper, and the grid containing the NP samples as a dry film was observed by TEM (FEI Tecnai) at an accelerating voltage of 120 kV.

#### Thermal analysis (TG/DTA) of ATA NPs

Thermal analyses that include thermogravimetry (TG) and differential thermal analysis (DTA) were performed using a Diamond TG/DTA (Perkin Elmer instrument)[37]. The precision of the thermobalance in this instrument is 0.0001 mg. Before performing the experiments, the weight, temperature, and sensitivity calibrations of the instrument were performed using calibration sets. The thermal decomposition was conducted on free ATA, F68, mPEG-PLGA, a physical mixture of the three compounds, and freeze-dried ATA NPs. Approximately 2 mg of a vacuum-dried coal sample of < 200 μm was placed in the sample crucible. The sample was heated in pure N_2_ from 30 to 400°C at a heating rate of 10°C/min. Melting points of the samples were determined from the resultant thermograms.

#### Storage stability and compatible stability of ATA NPs

To determine their storage stability, ATA NPs were diluted in phosphate buffered saline (PBS, pH 7.4) and incubated at 4°C or 37°C for 1-7 days. The size and zeta potential of the NPs were measured at designated intervals during this period. To measure the compatibility-stability, ATA NPs were added into solutions of 0.9% NaCl or 5% glucose, and incubated at 25°C for 24 h. From all conditions, 1 mL of suspension was collected for analysis of size and zeta potential.

#### Drug release from ATA NPs

The release of ATA from ATA NPs was investigated using dialysis and compared with the release of free ATA in solution [38–41]. Free ATA, or ATA NPs containing an equal amount of ATA, was mixed with release medium (6 mL ultra-pure water with 1% SDS) and placed in a dialysis sack (MWCO 12,000, Sigma). The pocket was then immersed in release medium (50 mL) with gentle stirring at 37°C. Samples of medium from outside of the pocket (2 mL) were taken at the designated time intervals for analysis, and an equal volume of fresh release medium was replenished. The amount of released drug was determined by the RP-HPLC method described in the section “*Pharmacokinetics of ATA NPs in rats*” below. The similarity of release profiles between the free ATA and ATA NPs were evaluated by the similarity factor (f2) shown below:

$$f2=50\log\;\left\{{\left[1+\frac{1}{n}\sum_{t=1}^{n}{\left(Rt-Tt\right)}^{2}\right]}^{-0.5}\times 100\right\}$$

where n is the number of the time points, *Rt* is the release value of ATA NPs at time *t*, and *Tt* is the release value of free ATA.

### Toxicity evaluation of ATA NPs

#### Effects of free ATA and ATA NPs on cell viability of MCF-7 cells and HUVEC

The MTT reduction ability was determined as an index of the metabolic viability, in particular mitochondrial function, of the cells. Briefly, MCF-7 cells or HUVEC were seeded in 96-well plates and allowed to grow overnight. Free ATA (dissolved in DMSO, diluted with DMEM), ATA NPs, or blank NPs were added at different concentrations and the cells were incubated for 24, 48, and 72 h. MTT solution (10 μL; 5 mg/mL) was added into each well for 4 h. One hundred microliters of 1% SDS (containing 0.1% concentrated hydrochloric acid) was then added in each well to dissolve the formazan crystals. After 8 h, the absorbance of solubilized MTT formazan products were measured at 595 nm, and the cell viability was calculated from the OD value of the test sample and the vehicle control (untreated cells) using the following equation: cell viability (%) = OD_test sample_ /OD_vehicle control_ × 100. The data obtained in triplicate from three independent experiments were presented as the mean ± SD.

#### Effects of varying concentrations of free ATA and ATA NPs on development and heart rate of zebrafish larvae

Male and female zebrafish were bred in separate tanks. The temperature of water in the tanks was maintained at 28.5°C and pH 7.2-7.4. After mating, the selected embryos were collected at 4 hpf and placed in 96-well plates. The chorion surrounding the embryo was removed enzymatically at 4 hpf following procedures described in the literature [42–43]. In brief, these embryos were placed in glass petri dishes containing of fresh water (25 mL) with pronase (50 μL; 50 mg/mL) for 4-5 min. Next, the fresh water was decanted and replenished with fresh E3 zebrafish embryos medium (13.7 mM NaCl, 0.54 mM KCl, 0.025 mM Na_2_HPO_4_, 0.044 mM KH_2_PO_4_, 0.42 mM NaHCO_3_, 1.3 mM CaCl_2_, 1.0 mM MgSO_4_, and 0.35 mM penicillin and streptomycin; pH adjusted to 7.2).

Various concentrations of free ATA and ATA NPs were added. The temperature of incubator was maintained at 28.5°C to ensure the optimal development of the embryos. Throughout the experiment, incubation solutions with freshly added ATA were replaced daily. Images of the zebrafish were captured in order to document any morphological changes. The heart rate was also recorded as described previously [44–47]. Twenty zebrafish were used in each experimental group and the zebrafish experiments were conducted at least four times.

### Pharmacokinetics of ATA NPs in rats

#### Pharmacokinetics of ATA NPs in rats

Female SD rats (INVIVOS, Singapore) were randomly divided into two groups (n = 6): free ATA or ATA NPs. Free ATA was suspended in a solution of PEG300: Ethanol: Tween 80 (60:25:15, v:v:v). Free ATA suspension or ATA NPs were administered at an equivalent dose of ATA at 8 mg/kg body weight via a single intravenous injection in the tail vein. At different time points (15 min, 30 min, 1, 2, 3, 4, 5, 6, 8, and 24 h), blood samples (0.3 mL) were collected from the suborbital vein and put into heparinized tubes. The blood samples were centrifuged at 3,000 rpm at 4°C for 10 min to obtain plasma. Then, the samples were pretreated and injected into RP-HPLC for detection [30–34]. Microsoft Excel PKSolver was used to analyze the pharmacokinetic data. All animal studies were done in accordance with the protocol of ARF SBS NIE A-0146 AZ approved by the Institutional Animal Care and Use Committee, Nanyang Technological University, Singapore.

#### Sample pretreatment

Before the samples were injected into the chromatograph, they were subjected to protein denaturation and precipitation [32–36]. Briefly, acetonitrile (700 μL) was added to each 300 μL sample to precipitate the proteins and extract ATA. Samples were vortex mixed for 15 min and then centrifuged for 10 min at 14,000 rpm at 4°C. The deproteinized supernatant was recovered and analyzed immediately by RP-HPLC.

#### Analytical method for determination of ATA in rat plasma and blood

The concentration of ATA in plasma or whole blood was measured by RP-HPLC analysis as described above. The standard stock solution of ATA was added into the plasma or blood at different concentrations and subjected to sample pretreatment. The calibration graphs were based on linear regression analysis of the peak-area of ATA versus the concentration of ATA [32–36]. The extraction recoveries of ATA from rat plasma were determined at the concentrations of 0.0005, 0.01, and 50 μg/mL and from rat blood at the concentrations of 0.1, 5, and 100 μg/mL, using five replicates. The precision and accuracy of the method were examined at three concentrations of samples. The intra-day precisions were evaluated by analyzing five replicate samples in one day. The analysis was performed on five consecutive days for the inter-day precision.

### Statistical analysis

Statistical analysis was performed using SPSS 10.0 software. Descriptive data were expressed as the arithmetic mean value plus or minus the standard deviation. All quantitative results were obtained from a minimum of triplicate samples. A *t*-test was applied to detect differences between groups. In all evaluations, *p < 0.05 was considered statistically significant.
